# Cognitive training and brain stimulation in patients with cognitive impairment: a randomized controlled trial

**DOI:** 10.1186/s13195-024-01381-3

**Published:** 2024-01-11

**Authors:** Daria Antonenko, Anna Elisabeth Fromm, Friederike Thams, Anna Kuzmina, Malte Backhaus, Elena Knochenhauer, Shu-Chen Li, Ulrike Grittner, Agnes Flöel

**Affiliations:** 1 Department of Neurology, Universitätsmedizin Greifswald, Ferdinand-Sauerbruch-Straße, 17475 Greifswald, Germany; 2 Chair of Lifespan Developmental Neuroscience, Technische Universität Dresden, 01062 Dresden, Germany; 3 Centre for Tactile Internet With Human-in-the-Loop, Technische Universität Dresden, 01062 Dresden, Germany; 4 Berlin Institute of Health (BIH), 10187 Berlin, Germany; 5 Institute of Biometry and Clinical Epidemiology, Charité – Universitätsmedizin Berlin, Humboldt-Universität Zu Berlin, Berlin Institute of Health, 10117 Berlin, Germany; 6 German Centre for Neurodegenerative Diseases (DZNE) Standort Greifswald, 17475 Greifswald, Germany

**Keywords:** Transcranial direct current stimulation, Mild cognitive impairment, Subjective cognitive decline, Electric field simulation, Resting-state functional connectivity

## Abstract

**Background:**

Repeated sessions of training and non-invasive brain stimulation have the potential to enhance cognition in patients with cognitive impairment. We hypothesized that combining cognitive training with anodal transcranial direct current stimulation (tDCS) will lead to performance improvement in the trained task and yield transfer to non-trained tasks.

**Methods:**

In our randomized, sham-controlled, double-blind study, 46 patients with cognitive impairment (60–80 years) were randomly assigned to one of two interventional groups. We administered a 9-session cognitive training (consisting of a letter updating and a Markov decision-making task) over 3 weeks with concurrent 1-mA anodal tDCS over the left dorsolateral prefrontal cortex (20 min in tDCS, 30 s in sham group). Primary outcome was trained task performance (letter updating task) immediately after training. Secondary outcomes included performance in tasks testing working memory (N-back task), decision-making (Wiener Matrices test) and verbal memory (verbal learning and memory test), and resting-state functional connectivity (FC). Tasks were administered at baseline, at post-assessment, and at 1- and 7-month follow-ups (FU). MRI was conducted at baseline and 7-month FU. Thirty-nine participants (85%) successfully completed the intervention. Data analyses are reported on the intention-to-treat (ITT) and the per-protocol (PP) sample.

**Results:**

For the primary outcome, no difference was observed in the ITT (*β* = 0.1, 95%-CI [− 1.2, 1.3, *p* = 0.93] or PP sample (*β* =  − 0.2, 95%-CI [− 1.6, 1.2], *p* = 0.77). However, secondary analyses in the N-back working memory task showed that, only in the PP sample, the tDCS outperformed the sham group (PP: % correct, *β* = 5.0, 95%-CI [− 0.1, 10.2], *p* = 0.06, d-prime *β* = 0.2, 95%-CI [0.0, 0.4], *p* = 0.02; ITT: % correct, *β* = 3.0, 95%-CI [− 3.9, 9.9], *p* = 0.39, d-prime *β* = 0.1, 95%-CI [− 0.1, 0.3], *p* = 0.5). Frontoparietal network FC was increased from baseline to 7-month FU in the tDCS compared to the sham group (*p*_FDR_ < 0.05). Exploratory analyses showed a correlation between individual memory improvements and higher electric field magnitudes induced by tDCS (*ρ*_tDCS_ = 0.59, *p* = 0.02). Adverse events did not differ between groups, questionnaires indicated successful blinding (incidence rate ratio, 1.1, 95%-CI [0.5, 2.2]).

**Conclusions:**

In sum, cognitive training with concurrent brain stimulation, compared to cognitive training with sham stimulation, did not lead to superior performance enhancements in patients with cognitive impairment. However, we observed transferred working memory benefits in patients who underwent the full 3-week intervention. MRI data pointed toward a potential intervention-induced modulation of neural network dynamics. A link between individual performance gains and electric fields suggested dosage-dependent effects of brain stimulation. Together, our findings do not support the immediate benefit of the combined intervention on the trained function, but provide exploratory evidence for transfer effects on working memory in patients with cognitive impairment. Future research needs to explore whether individualized protocols for both training and stimulation parameters might further enhance treatment gains.

**Trial registration:**

The study is registered on ClinicalTrials.gov (NCT04265378). Registered on 7 February 2020. Retrospectively registered.

**Supplementary Information:**

The online version contains supplementary material available at 10.1186/s13195-024-01381-3.

## Background

The development of new treatment options for cognitive impairment associated with older age is urgently needed. Repeated sessions of training and non-invasive brain stimulation (NIBS) have the potential to enhance cognition in patients with mild cognitive impairment (MCI) which presents a transitional stage between healthy aging and dementia, e.g., due to Alzheimer’s disease (AD) [1]. Mild forms of cognitive impairments usually start several years before the clinical diagnosis of dementia and include subjective cognitive decline (SCD) [2] and MCI. During these early phases of AD, the application of non-pharmacological therapeutic interventions may decelerate the neurodegenerative process, preventing dementia stages for as long as possible [3–5].

In this context, pairing a cognitive training intervention with an NIBS technique such as transcranial direct current stimulation (tDCS) has been suggested to be an attractive, safe, and beneficial treatment option [4]. Combined with training, tDCS increases cortical excitability by changing membrane potentials toward depolarization, tuning ongoing neural processes, and promoting long-term-potentiation-like synaptic plasticity [6]. Previous studies have used tDCS as a non-invasive and safe method of electrical brain stimulation which may induce longer-lasting functional benefits, particularly if applied in concurrence with intense task practice over multiple days [6, 7]. Specifically, studies have reported enhanced working memory functions in young and older adults through stimulating the dorsolateral prefrontal cortex (DLPFC) during executive control training [8–10]. Beyond immediate effects of the intervention, some studies have observed partially sustained improvements in working memory functions in healthy adults [11–13]. In patients with cognitive impairment and dementia due to AD, reports of beneficial effects remain limited and heterogeneous [4, 14, 15]. Two randomized controlled trials in patients with early stages of cognitive impairment (such as MCI or mild AD) have reported improved cognitive performance after combined tDCS and training interventions [16, 17], while two others did not find intervention benefits [18, 19].

In studies involving concurrent measures of neural activity and connectivity, tDCS reduced network deficiencies in MCI [20] and was discussed to potentially delay the neuropathological disease progression by increasing release of brain-derived neurotrophic factor, or boosting β-amyloid clearance from the brain [21, 22]. In healthy adults, modulation of functional connectivity (FC) in specific cognition-relevant networks has been observed to result from tDCS application over task-relevant brain areas [23–25]. Resting-state functional resonance imaging (fMRI) before, during, or after prefrontal anodal tDCS and working memory training have revealed FC increases within the targeted frontoparietal network in young and older adults [11, 25–27]. The findings of a previous trial, performed on healthy older adults, showed beneficial effects of a multisession cognitive training combined with prefrontal tDCS on near-transfer task performance which persisted for a month after the intervention [28], as well as alterations in prefrontal FC and microstructural integrity [29]. Additionally, evidence using computational modeling of electric fields has pointed toward a potential link between individual tDCS-induced field magnitudes and behavioral and neurophysiological tDCS effects [28, 30, 31].

Based on this evidence observed in healthy older adults, we performed a randomized, phase II, sham-controlled, double-blind clinical study to investigate the efficacy of a multisession cognitive training combined with tDCS in patients with SCD or MCI. Participants were randomly assigned to one of two intervention groups (target intervention: anodal tDCS over the left DLPFC, control intervention: sham tDCS). Potential effects on behavioral performance were evaluated on trained and non-trained tasks at both immediate (3-day post) and delayed time points (1- and 7-months follow-up). With complementary MRI assessments before and 7 months after the intervention (i.e., follow-up assessment), we opted to investigate the underlying neural mechanisms with respect to stimulation-induced functional connectivity modulations and the potential linkage to magnitudes of the elicited electric fields which could vary between participants.

## Methods

### Study design and participants

In this monocenter, double-blind randomized controlled trial, we compared cognitive training with concurrent tDCS (target intervention) to cognitive training with sham stimulation (control intervention). Both groups underwent nine sessions that were evenly distributed over 3 weeks (see below for details on study population). The study was performed at University Medicine Greifswald. Eligibility criteria comprised age between 60 and 80 years, right-handedness, presence of SCD or MCI [2, 32], exclusion criteria history of neurological (e.g., epilepsy, seizures, strokes), or neurodegenerative disorders (e.g., dementia), severe and untreated medical conditions, history of severe alcoholism, or use of drugs and severe psychiatric disorders (e.g., psychosis). The full study protocol, including eligibility criteria, detailed descriptions of the tasks, and statistical analysis plan, has been published previously [33]. Participants were recruited via advertisement in newspapers and from the local memory clinic. The study protocol was approved by the ethics committee of the University Medicine Greifswald, conducted in accordance with the Helsinki Declaration, and registered at ClinicalTrials.gov (Identifier NCT04265378). All participants gave written informed consent (see Additional file 1: Table S1 for baseline characteristics of the study sample).

### Randomization and masking

Sample size calculations were published in the study protocol [33]. Estimating an effect size of 0.85 to demonstrate an effect in the primary outcome, 46 participants had to be included in the analysis with an independent *t*-test using a two-sided significance level of 0.05 and a power of 80%. Forty-six eligible participants were randomly allocated to target and control intervention groups, stratified by age (cut-off, 70 years) and baseline performance on the trained letter updating task (cut-off, two lists) [33]. Randomization blocks with varying block sizes were generated for each of the four groups, using R software (http://www.R-project.org) and the blockrand package (https://CRAN.R-project.org/package=blockrand). The researcher who generated the allocation, enrolled participants and assigned participants to the groups was unaware of the stimulation condition. Moreover, the researchers conducting the experiment were unaware of group assignment. Participant blinding was ensured using sham stimulation in the control intervention group: the current was initially applied for 30 s to elicit the typical tingling sensation of active stimulation on the scalp and subsequently turned off. Previous research has shown that sham tDCS is a valid and safe method for blinding participants [34]. After the last training session, participants were asked to state whether they believed they had received anodal or sham tDCS.

### Procedures

#### Cognitive training and transfer tasks

In each of the nine cognitive training visits, two cognitive training tasks (letter updating task [35] and three-stage Markov decision-making task [36]) were administered. Participants first performed the letter updating task on a tablet, where 15 lists of the letters A to D (varying in their length between 5 and 13 letters) were presented in a random order. After each list, participants were asked to recall the last four letters. The second training task was a three-stage Markov decision-making task on a laptop where participants had to learn to choose the optimal sequence of actions to maximize overall gains and minimize overall loss over two learning conditions (immediate and delayed reward) that differed in their action-outcome associations (equal vs. variable over the three stages).

At pre-, post-, and follow-up assessments, the three transfer tasks (near-transfer for letter updating task: N-back task, near-transfer for Markov decision-making task: Wiener matrices test [37], far transfer: Auditory Verbal Learning Test (AVLT) [38, 39]) were administered after the training tasks. First, a numerical N-back task (of varying load, i.e., a 1-back and a 2-back condition, with nine trials of ten items each) was performed. Next, the German version of the AVLT was administered, where a list of 15 words had to be learned over five blocks. In the 30-min interval before the delayed recall of the word list, participants performed the Wiener Matrices Test (WMT-2) that required selecting a target among distractors that completes a figural matrix (18 different matrices were presented). Parallel versions of the tasks were administered in a counterbalanced order for each session.

#### Transcranial direct current stimulation

Cognitive training was administered while participants received either anodal or sham tDCS via a battery-operated stimulator (NeuroCare Group GmbH, Munich, Germany). At the beginning of each session, the tDCS setup was mounted with two saline-soaked sponge electrodes (5 × 7 cm each; anode centered over F3, cathode centered over the contralateral supraorbital cortex) using the 10–20 EEG-system grid. Direct current was delivered with 1 mA intensity for 20 min in the tDCS group and for 30 s in the sham group (10 s fade in/out). The stimulation started simultaneously with the letter updating task and finished after approximately the first half of the Markov task. Adverse events were assessed by questionnaire at the end of every third training session [34].

### Outcomes

#### Primary outcome

The primary outcome was performance on the letter updating task (measured by number of correctly recalled lists) at post-assessment, which reflects working memory function.

#### Secondary outcomes

##### Performance measures

Secondary outcomes were performance in decision-based learning at post-assessment as measured by the proportion of optimal actions in the delayed condition of the Markov decision-making task as well as working memory (letter updating) and decision-based learning performance at follow-up assessments. Other secondary outcomes were performances on the transfer tasks at post and follow-up assessments: near-transfer was measured by percent correct working memory performance and d-prime in the N-back task. Far-transfer tasks included the German version of the AVLT which measures episodic memory performance by the number of words recalled at delayed recall, and the WMT-2 Test which assesses reasoning ability (percent of matrices completed correctly).

##### MRI data acquisition

MR images were acquired at the Baltic Imaging Center (Center for Diagnostic Radiology and Neuroradiology, University Medicine Greifswald) on a 3-T Siemens Verio scanner using a 32-channel head coil. Resting-state fMRI scans were acquired using an echo-planar-imaging sequence (3 × 3 × 3 mm^3^ voxel size, repetition time (TR) = 2000 ms, echo time (TE) = 30 ms, flip angle = 90°, 34 slices, descending acquisition, field of view 192 × 192 mm^2^, 176 volumes, TA = 6.00 min). Participants were instructed to keep their eyes closed, to not think of anything in particular, and to try not to fall asleep (whether participants fell asleep or not was assessed per self-report directly after the resting-state scan; no participant reported having fallen asleep). High-resolution anatomical images were acquired using three-dimensional T1-weighted magnetization prepared rapid gradient echo imaging (1 mm ^3^ isotropic voxel, TR = 2300 ms, TE = 2.96 ms, inversion time = 900 ms, flip angle = 9°, 256 × 240 × 192 mm^3^ matrix). Furthermore, a diffusion weighted spin-echo echo-planar imaging sequence was acquired (1.8 × 1.8 × 2.0 mm^3^ voxel size, TR = 11,100 ms, TE = 107 ms, 70 slices, 64 directions (b = 1000 s/mm^2^), 1 b0).

##### Functional connectivity

Resting-state fMRI data were analyzed using the CONN toolbox (www.nitrc.org/projects/conn) [40] and SPM [41], with all settings chosen as in our previous study with healthy older adults [29].

Functional and anatomical data were preprocessed using a flexible preprocessing pipeline [42] including realignment with correction of susceptibility distortion interactions, slice-timing correction, outlier detection, direct segmentation and MNI-space normalization, and smoothing. Functional data were realigned using the SPM realign and unwarp procedure [43], where all scans were coregistered to a reference image (first scan of the first session) using a least squares approach and a 6-parameter (rigid body) transformation [44], and resampled using b-spline interpolation to correct for motion and magnetic susceptibility interactions. Temporal misalignment between different slices of the functional data (acquired in descending order) was corrected following SPM slice-timing correction (STC) procedure [45], using sinc temporal interpolation to resample each slice BOLD timeseries to a common mid-acquisition time. Potential outlier scans were identified using ART [46] as acquisitions with framewise displacement above 0.9 mm or global BOLD signal changes above 5 standard deviations [47], and a reference BOLD image was computed for each subject by averaging all scans excluding outliers. Functional and anatomical data were normalized into standard MNI space, segmented into gray matter, white matter, and CSF tissue classes, and resampled to 2-mm isotropic voxels following a direct normalization procedure [48, 49] using the SPM unified segmentation and normalization algorithm [50, 51] with the default IXI-549 tissue probability map template. Finally, functional data were smoothed using spatial convolution with a Gaussian kernel of 6 mm full-width half-maximum (FWHM).

In addition, functional data were denoised using a standard denoising pipeline including the regression of potential confounding effects characterized by white matter timeseries (5 CompCor noise components), CSF timeseries (5 CompCor noise components), motion parameters and their first-order derivatives (12 factors) [52], outlier scans (below 94 factors) [47], session effects and their first-order derivatives (2 factors), and linear trends (2 factors) within each functional run, followed by high-pass frequency filtering of the BOLD timeseries [53] above 0.01 Hz. CompCor [54] noise components within white matter and CSF were estimated by computing the average BOLD signal as well as the largest principal components orthogonal to the BOLD average, motion parameters, and outlier scans within each subject’s eroded segmentation masks. From the number of noise terms included in this denoising strategy, the effective degrees of freedom of the BOLD signal after denoising were estimated to range from 135.4 to 288 (average 231.9) across all subjects [48].

Seed-based connectivity maps were estimated characterizing the patterns of functional connectivity using the Harvard–Oxford atlas ROIs [55]. Functional connectivity strength was represented by Fisher-transformed bivariate correlation coefficients from a weighted general linear model (weighted-GLM), defined separately for each pair of seed and target areas, modeling the association between their BOLD signal timeseries. Individual scans were weighted by a boxcar signal characterizing each individual session convolved with an SPM canonical hemodynamic response function and rectified.

Group-level analyses were performed using a general linear model (GLM) [42] with a 2 (groups: anodal, sham) × 2 (time points: pre, FUII) design. The interaction between group and time point was assessed to examine whether functional connectivity alterations from pre to FU differed between the anodal and sham groups. Age and sex were included as covariates. For each individual voxel, a separate GLM was estimated, with first-level connectivity measures at this voxel as dependent variables (one independent sample per subject and one measurement per session), and groups as independent variables. Voxel-level hypotheses were evaluated using multivariate parametric statistics with random-effects across subjects and sample covariance estimation across multiple measurements. Inferences were performed at the level of individual clusters (groups of contiguous voxels). Cluster-level inferences were based on parametric statistics from Gaussian Random Field theory [56]. The results were thresholded using a combination of a cluster-forming *P* < 0.001 voxel-level threshold, and a familywise-corrected *P*_FDR_ < 0.05 cluster-size threshold [57].

##### Microstructural and volumetric analyses

T1 and DTI data were processed by FreeSurfer version 7 (https://surfer.nmr.mgh.harvard.edu) [58] and FSL version 6 (https://fsl.fmrib.ox.ac.uk/fsl/fslwiki) [59]. First, T1 data were processed by the FreeSurfer’s cross-sectional pipeline (recon-all) which includes motion correction, skull stripping, normalization, intensity correction, volumetric segmentation, and cortical surface reconstruction [60]. Second, the longitudinal pipeline was applied to create a robust, unbiased which-subject template using robust, inverse consistent registration which increases reliability and statistical power, for the detection of brain structural changes that may occur with intervention [58, 61]. Quality assessment involved visual inspection of all processing steps and calculation of anatomical signal to noise ratios using FreeSurfer QAtools https://github.com/Deep-MI/qatools-python). All structural data were deemed appropriate for analysis. Regional volumes were extracted for the ROI corresponding to the stimulation target (i.e., left middle frontal gyri from the Desikan-Killiani atlas [62]) and adjusted for total intracranial volume.

DTI data preprocessing included eddy current and head motion correction using an automated affine registration algorithm. A diffusion tensor model was fitted to the motion-corrected DTI data at each voxel to create individual 3-dimensional FA and MD maps. Probabilistic fiber tracking was conducted in FSL; this method repeatedly samples the distribution at each voxel to produce “streamlines” that connect voxels from selected seed regions. The following parameters were applied: 5000 streamline samples, 0.5 mm step length, and curvature threshold = 0.2. The left middle frontal gyrus from the Harvard–Oxford atlas used for resting-state fMRI analyses, transformed into individual DTI space, multiplied with diffusion maps and binarized, was used as seed regions for the tracts [63]. Given the large size and extent of prefrontal streamlines, paths were thresholded by 10% of the individual tract-specific connection probability to reduce the likelihood of including extraneous tracts [64]. The mean FA for all streamlines was then calculated by masking the tracts with individual diffusion maps, binarizing to define tract masks, and averaging individual voxel values along the tract which was then entered into statistical analyses.

Individual T1-weighted images were coregistered to the b0 images, using rigid-body transformation. These registrations were used to transform masks of the left stimulation target to the MD maps. To extract MD from the gray matter within the stimulation target, the individually segmented left middle frontal gyrus was masked by the ROI used for seed-based tractography and resting-state FC analyses, in line with previous studies [65].

##### Electric field simulations

The software SimNIBS version 4 (simnibs.org) [66, 67] was used to build the head models and electric field simulations, using default conductivity parameters implemented in the toolbox. A finite element mesh was generated from T1- and T2-weighted images, including representations of the scalp, skull, spongy bone, cerebrospinal fluid, gray matter, and white matter. Head models were inspected for quality assessment of head segmentation, resulting in the exclusion of two poor-quality head meshes (final sample for simulation analyses: *n* = 39). The 90th percentile of the electric field magnitude over the whole cortical surface was estimated.

### Statistical analysis

The predefined analyses were conducted using IBM SPSS software (version 29) and R (v4.2.1) [68] as described in the statistical analysis plan, which was uploaded before the analysis of the primary outcome. All participants who received at least 1 day of intervention were included in the full dataset for intention-to-treat analysis. Multiple imputation by chained equations was performed with 30 imputed datasets using predictive mean matching to estimate missing values. The per-protocol (PP) analysis set comprises all participants who completed all nine visits of the 3-week intervention. Separate linear mixed model analyses were conducted for each task, for post-assessment and follow-up time points, adjusted for age and baseline scores (see Supplementary Methods). We report model-based marginal means and group differences with 95% confidence intervals (CIs). Spearman correlation coefficients were computed as association measures between performance effects and modeling-based electric field strengths. A two-sided significance level of *α* = 0.05 was used.

## Results

**Fig. 1:**
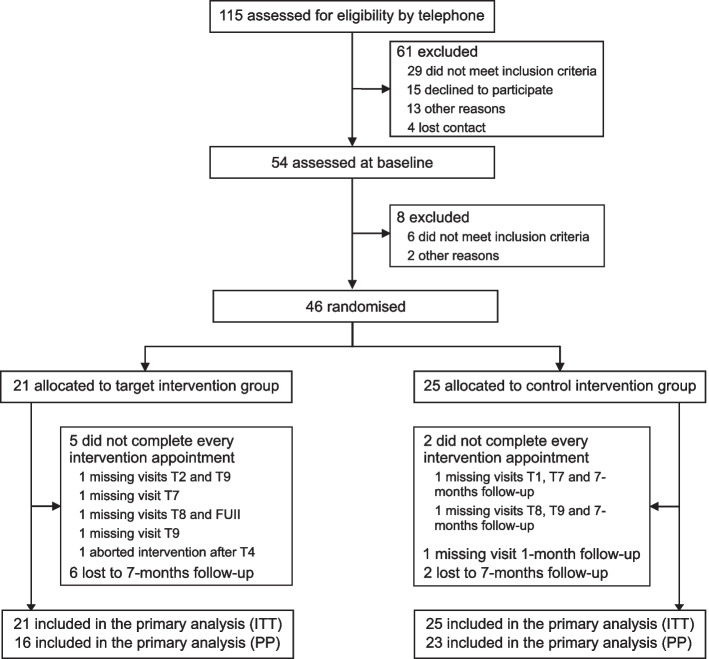
Consolidated Standards of Reporting Trials (CONSORT) diagram. Intention-to-treat analysis (ITT) was performed for the primary outcome at post-assessment (*N* = 46). Seven participants did not receive the complete intervention and were therefore not included in the per-protocol analysis (PP, *n* = 39)

From May 17, 2019, to November 25, 2021, we screened 115 potential participants, of whom 54 were invited for baseline assessment and 46 (18 female) underwent randomization (Fig. 1). The mean age of the total sample was 69.8 years (*SD* = 5.1, age range 60–80 years). The last post-assessment (primary outcome) was completed on February 7, 2022, and the last 7-month follow-up was completed on October 10, 2022. Seven participants did not complete the whole intervention due to illness, resulting in a per-protocol study sample of *n* = 39 (tDCS: *n* = 16 (6 females), mean/SD age 70.0/5.2 years; sham: *n* = 23 (9 females), mean/SD age 69.8/4.6 years).

### Behavioral task performance

Statistical analyses of the full ITT dataset revealed no substantial treatment effects on any tested secondary behavioral task performance parameter (see Additional file 1: Figure S1). However, marginal effects for the 7-month follow-up assessment revealed slightly superior performance in the N-back task (d-prime) of the anodal group compared to the sham group (adjusted mean [95%-CI] in the anodal group: 2.2 [2.0–2.5], adjusted mean [95%-CI] in the sham group: 1.9 [1.6–2.1]; *P* = 0.06).

**Fig. 2:**
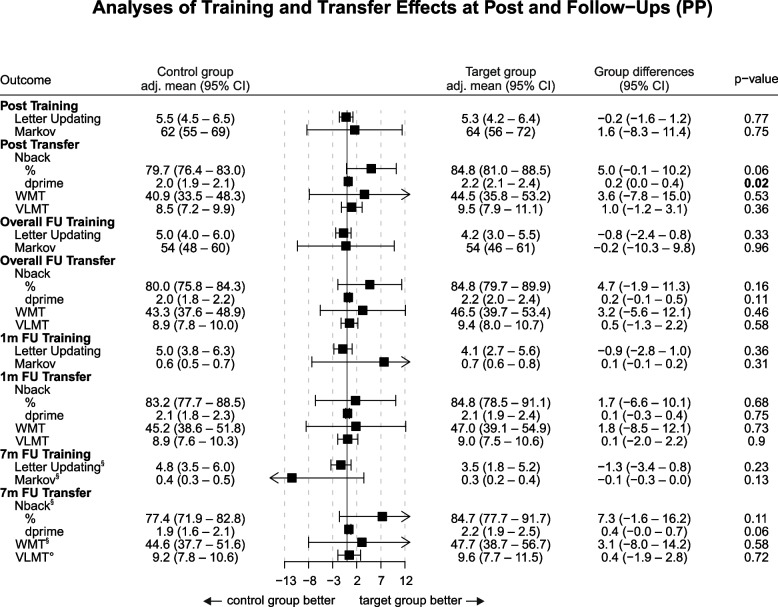
Forest plot for performance outcomes (per-protocol set). Per-protocol analyses of training and transfer effects at post and follow-ups. Abbreviations and units: Letter Updating # correct. Markov %, optimal actions. Nback % correct and d-prime. WMT % correct. VLMT (German version of the AVLT) # words recalled. Separate linear mixed model analyses were conducted for post-assessment and follow-up time points, for each task (i.e., 1/7mFU values are derived from the same models as for the corresponding overall FU scores). In the case of missing data, the results are based on multiple imputation. For separate time points: *n* = 39 if not indicated otherwise. ^§^*n* = 34. °*n* = 33. AVLT, auditory verbal learning test; CI, confidence interval; FU, follow-up; WMT, Wiener Matrices Test

Model-derived adjusted means per group and adjusted treatment effects (group differences) for all training and transfer tasks in the per-protocol sample are shown in Fig. 2.

**Fig. 3:**
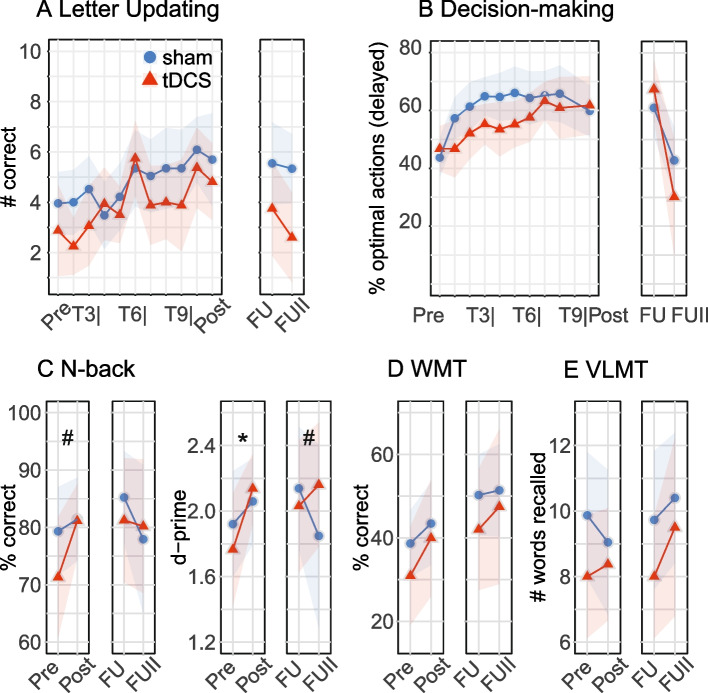
Training and transfer task performance (per-protocol set). **A** Training improvement in the letter updating task. **B** Training improvement in the Markov decision-making task. No enhanced training gains were observed after anodal stimulation compared to sham stimulation. **C** Enhanced performance in the N-back task after anodal stimulation compared to sham stimulation. **D** Transfer task performance in the WMT. **E** Transfer task performance in the VLMT. There were no differences in WMT or VLMT between anodal and sham groups. Pre, pre-assessment. T3, T6, T9, training days 3, 6, 9. Dots represent mean values and shaded areas indicate 95% confidence intervals. tDCS, transcranial direct current stimulation. FU, 1-month follow-up. FUII, 7-months follow-up. WMT, Wiener Matrices Test. VLMT, verbal learning and memory test

Figure 3 displays mean performance scores on all behavioral tasks for the PP dataset. For the primary outcome letter updating performance at post-assessment, no substantial treatment effect emerged (Fig. 3a).

For the per-protocol set, no group differences at post-assessment were observed for the Markov decision-making task and no sustained effects emerged at follow-up for either training task (Fig. 3b). For the N-back task, the anodal group improved compared to the sham group at post-assessment, with this difference being more pronounced in d-prime than in % correct values (mean difference for % correct [95%-CI] 5.0 [− 0.1–10.2], *P* = 0.06, Cohen’s *D* = 0.62; d-prime 0.2 [0.0–0.4], *P* = 0.02, Cohen’s *D* = 0.78; Fig. 3c). These observed effects on N-back performance exceeded the “minimal clinical important differences” (MCID, computed by multiplying the pooled baseline SD with 0.2: for % correct = 3.6; for d-prime 0.14) [69, 70]. Analyses of follow-up effects showed a small (but not significant) difference in N-back d-prime values in the direction of a sustained performance enhancement in tDCS compared to the sham group (mean difference [95%-CI] 0.2 [− 0.1–0.5], *P* = 0.11). This difference was more pronounced at the 7-month follow-up (mean difference [95%-CI] 0.4 [0.0–0.7, *P* = 0.06]. For the transfer tasks WMT and VLMT, no substantial group differences emerged either at post-assessment or at follow-up (Fig. 3d and e).

### Seed-based functional connectivity

**Fig. 4:**
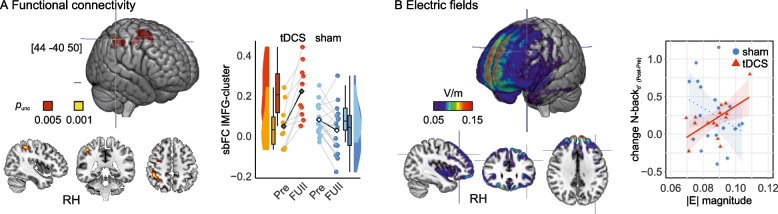
**A** Functional connectivity. Resultant cluster (*P*_FDR_ < 0.05; *P*_unc_ < 0.001 in yellow, *p*_unc_ < 0.005 in red) from seed-to-voxel resting-state FC analysis with seed in stimulation target (lMFG). Cluster location in the right supramarginal/angular gyrus (*x* = 44, *y* =  − 40, *z* = 50) and in the right superior/middle frontal gyrus (*x* = 22, *y* =  − 4, *z* = 60): increase in FC to the stimulation target in the anodal group compared to the sham group. Means (black diamonds for anodal and white diamonds for sham) and individual datapoints (single circles in orange/red for anodal and light blue/dark blue for sham). Box plots indicate median (middle line), 25th, 75th (box), and 5th and 95th percentile (whiskers). *N* = 27 independent participants. sbFC, seed-based functional connectivity. lMFG, left middle frontal gyrus. tDCS, transcranial direct current stimulation. RH, right hemisphere. **B** Computational modeling of electric fields. Group average of electric fields induced by anodal tDCS (in V/m), projected in “fsaverage” space. Scatterplots display the correlation between electric field magnitudes and change in N-back task performance (Post minus Pre of d-prime values), anodal: *ρ*_tDCS_ = 0.6, *P* = 0.02; sham: *ρ*_sham_ =  − 0.24, *P* = 0.31. Note that sensitivity analysis for the anodal group without the outlier yielded similar Spearman’s correlation coefficient (*ρ*_tDCS_ = 0.5, *P* = 0.05)

A subgroup of 27 participants (anodal: *n* = 10, sham: *n* = 17) completed the baseline as well as the 7-month follow-up MRI scan. Whole-brain seed-to-voxel analyses revealed a significant cluster in the right superior parietal lobe (i.e., supramarginal/angular gyrus; MNI coordinates: *x* = 44, *y* =  − 40, *z* = 50, |T(23)|> 3.77, *k* ≥ 80, cluster threshold: *P* < 0.05 cluster-size FDR corrected, voxel threshold: *P* < 0.001 uncorrected, Fig. 4a). A more lenient threshold of *P* < 0.005 supported that the cluster reflected connectivity within the frontoparietal executive control network (i.e., superior/middle frontal gyrus in the prefrontal cortex and supramarginal/angular gyrus in the posterior parietal cortex MNI coordinates: *x* = 44/22, *y* =  − 40/ − 4, *z* = 50/60, |T(23)|> 3.10, *k* ≥ 307, cluster threshold: *P* < 0.05 cluster-size FDR corrected, voxel threshold: *P* < 0.005 uncorrected). There was no correlation between the FC change and the working memory change (N-back task performance at 7-month FU minus at pre-assessment) due to the intervention (*ρ*_tDCS_ = 0.07, *P* = 0.88; *ρ*_sham_ =  − 0.03, *P* = 0.92).

### Microstructure and volume

No substantial differences emerged in the microstructure of white matter (WM) pathways (mean FA difference [95%-CI]: − 0.001 [− 0.014–0.011], *P* = 0.815), gray matter (GM) in the target region (mean MD difference [95%-CI]: − 5.3e − 05 [− 1.2e − 04–2.3e − 05], *P* = 0.163), or volumes of WM tracts (mean difference [95%-CI]: − 15.6 [− 397.5–366.2], *P* = 0.933) and GM targets (mean difference [95%-CI] 1.1e − 04 [− 1.9e − 04–4.2e − 04], *P* = 0.460) after the intervention between the anodal and sham stimulation groups (see Additional file 1: Table S2).

### Analyses of individually induced electric fields

Simulation of individual electric fields revealed stimulation of the frontal cortices induced by the applied electrode configuration and stimulation intensity (Fig. 4b). Higher-field magnitudes were associated with higher changes in d-prime scores of the N-back task in the anodal group (*ρ*_tDCS_ = 0.59, *P* = 0.016) but not in the sham group (*ρ*_sham_ =  − 0.24, *P* = 0.313; Fig. 4b).

### Adverse events and blinding

Fifteen adverse events were reported by 4 participants in the target group, and 16 adverse events were reported by 7 participants in the control intervention group. No serious adverse events occurred. The incidence of adverse events did not differ between groups (incidence rate ratio [95%-CI]: 1.1 [0.5, 2.2], Additional file 1: Table S3A). One participant (allocated to the anodal group) terminated participation due to dizziness in the 5th experimental session; dizziness receded completely within 2 h. The James Blinding index (mean [95%-CI]: 0.8 [0.7, 0.9], Additional file 1: Table S4) indicated overall blinding success. The Bang’s BI for the intervention group was 0.1 (95%-CI: − 0.2, 0.4) and − 0.3 (95%-CI: − 0.6, 0.02) for the control group, indicating blinding success within both groups.

## Discussion

We present the results of a 3-week intervention of cognitive training combined with anodal tDCS over the left DLPFC in patients with SCD or MCI. Overall, individuals in the target intervention group (i.e., anodal tDCS and training) did not outperform the individuals who received the control intervention (i.e., sham tDCS and training) in the trained letter updating task (primary outcome), indicating that the combined intervention did not lead to superior trained performance enhancement in patients with cognitive impairment. However, those individuals that completed all nine sessions of the intervention (PP sample) with tDCS compared to sham showed a superior improvement in a near-transfer N-back task (secondary outcome) immediately after the intervention. The intervention was safe and well-tolerated by our patients, producing only minor adverse effects, consistent with previous reports [28].

In this trial, the intervention did not prove beneficial for the primary outcome (trained letter updating performance immediately after the intervention), or any other outcome in the ITT sample. However, there was some indication of induced benefits for a secondary outcome assessing working memory performance (N-back task performance immediately after the intervention) in those individuals who received the full intervention (PP sample). This finding is consistent with our previous study where we applied the identical intervention in healthy older adults [28]; further supporting the idea that the benefit of tDCS combined with training may not be necessary in terms of enhancing training gains on the trained task (particularly if the training effect is large), but more in terms of enhancing the transfer effect. Other studies have likewise reported no additional benefits of tDCS in the trained task in older adults [10, 16, 18, 71] while an improvement in an untrained task was observed [16, 18]. In fact, several lines of evidence point toward tDCS-mediated induction of functional plasticity in untrained tasks within the same cognitive domain [6, 72]. For instance, a recent large-sampled trial applied adaptive N-back training over 4 weeks, and found tDCS-induced benefits on logical memory and word-list learning [16]. However, anodal tDCS in this study was administered to the lateral temporal cortex, so the findings may not be generalizable to other cortical sites or cognitive domains. Another trial in patients with MCI or dementia due to AD tested a 3-week intervention of multi-domain cognitive training with anodal tDCS over the DLPFC, and found improved working memory and attention scores [17]. Benefits persisted partly at 2 to 6 months [16, 17]. Moreover, previous studies have suggested that the N-back task may be particularly suitable to examine tDCS- and working memory-training-induced modulation [26, 73], possibly due to its reliable engagement of well-defined frontoparietal executive control networks [74]. Our findings corroborate these reports, extending the observations to other cognitive domains and stimulation parameters.

Furthermore, the inclusion of brain imaging data allowed us to elucidate the underlying neural mechanisms in cognitive impairment. Specifically, the underlying head and brain anatomy of individual subjects may be a key determinant of tDCS-induced effects [75]. Here, the total volume of the head, amount of cerebrospinal fluid or distance between electrodes and individual gray matter surface impact current flow and thus the magnitude of the electric field in the individual brain [75–77]. Due to age-related atrophy, older adults exhibit significantly lower electric field magnitudes [75, 77], a process probably even more pronounced in pathological aging [78]. In the current study, we found a link between electric field magnitudes and working memory performance with higher magnitudes related to superior behavioral tDCS effects, supporting previous results [30, 31] and extending them to patients with cognitive impairment. This finding lends further support to the concept of dose–response relationships on an individual level and may help to individually tailor brain stimulation approaches in the future, particularly in individuals with age-or disease-related brain atrophy [79].

Using resting-state fMRI data acquired before the intervention and at follow-up, we observed FC alterations in the frontoparietal network in the anodal group compared to the sham group. While our previous study with healthy older adults [29] reported post-MR data immediately after the 3-week intervention, here, we showed FC effects lasting 7 months after the intervention. Specifically, FC of the stimulation target in the left-hemispheric middle frontal gyrus with the angular gyrus and the superior/middle frontal gyrus in the right hemisphere, cortical areas known to be part of the frontoparietal executive control network, was increased [27]. Long-term modulation of functional coupling in this network was conceptually in line with immediate effects on resting-state FC seen after anodal tDCS over the DLPFC in older adults [25, 26]. In contrast, we did not observe any microstructural or volumetric effects, suggesting that long-term effects on the microstructural or anatomical level (in terms of white and grey matter integrity or volume) are not induced by our combined tDCS-plus-training approach over nine sessions. With regard to FC, our findings extend previous evidence and suggest that tDCS-induced plasticity at the level of functional coupling may be long-lasting in patients with SCD and MCI. However, as individual FC changes were not related to working memory benefits in our sample, their functional significance remains inconclusive and needs to be elucidated in future studies.

### Clinical relevance of working memory improvements

The clinical significance of the observed improvement in working memory functions is difficult to establish due to the relatively small study sample and the lack of daily-life-relevant outcome measures such as the “Activities of Daily Living for Mild Cognitive Impairment (ADCS-MCI-ADL)” scale [80] or the “Clinical Dementia Rating” (CDR) [81, 82]. However, the N-back task used as a transfer outcome measure is a well-established and reliable index of working memory, which presents a fundamental target of therapeutic interventions [83]. In order to further evaluate the potential clinical significance of the improvement, we have additionally computed the “minimal clinically important difference”, MCID [69, 70], which indicated that our observed effects might be clinically relevant. However, clinical significance of tDCS interventions in the context of patients with cognitive impairment still remains inconclusive [16], warranting longer training, boosting sessions, larger RCTs, and home-based approaches [15, 82].

### Limitations

Some limitations must be considered. First, the study included a relatively small sample of patients with SCD or MCI, tested at a single site. Due to the relatively high drop-out rate for MR assessments, the sample for imaging analysis was even smaller. However, multimodal assessment was conducted including several cognitive tasks and cognitive domains, multiple timepoints, and imaging parameters, allowing a comprehensive assessment of the effects and underlying mechanisms; despite the rather small sample size, we observed (moderate to) large effect sizes (Cohen’s *D* = 0.62 for % correct, Cohen’s *D* = 0.78 for d-prime). Nevertheless, future larger trials are required to evaluate the robustness of the effects. Second, we did not acquire AD biomarkers such as Tau and Aβ in our sample to provide information with regard to underlying pathology. However, we argue that for this particular treatment approach (tDCS-plus-training) which does not specifically target amyloid or tau, impairment of cognition and underlying neural networks is crucial, and efficacy is rather independent of the etiology of the disorder.

## Conclusions

Repeated sessions of cognitive training with concurrent brain stimulation did not lead to superior performance enhancements in patients with cognitive impairment. However, in individuals who underwent the full intervention, we observed benefits on a near-transfer working memory task. Functional connectivity increases in the frontoparietal network may have pointed toward a long-term modulation of neural network dynamics through the intervention, with clinical significance of the effects remaining however inconclusive. A link of individual gains with electric field magnitudes indicated a dose–response relationship. Future studies must explore the potential of individualized protocols, both for training and stimulation parameters, to advance the development of non-pharmacological interventions that enhance functions in patients with SCD and MCI, and possibly even halt disease progression.

### Supplementary Information

**Additional file 1:** **Table S1.** Baseline characteristics. **Figure S1.** Forest plot for performance outcomes (ITT sample). Intention-to-treat analyses of training and transfer effects at post and follow-ups. Abbreviations and units: Letter Updating # correct. Markov, % optimal actions. N-back, % correct and d-prime. WMT, % correct. VLMT (German version of the Auditory Verbal Learning Test, AVLT) # words recalled. Separate linear mixed model analyses were conducted for post-assessment and follow-up time points, for each task (i.e., 1/7-months FU values are derived from the same models as for the corresponding overall FU scores). In case of missing data, results are based on multiple imputation. FU, follow-up. WMT, Wiener Matrices Test. VLMT, verbal learning and memory test. For separate time points: *N* = 46 if not indicated otherwise. *n = 45. ^#^*n* = 44. ^§^*n* = 34. °*n* = 33. **Table S2.** Microstructural and volumetric analysis (MRI sample). **Table S3.** Self-reported incidence of adverse events (at least moderate symptoms). **Table S4.** Number of participants by group assignment and guess.

## Data Availability

The datasets of the current study are available from the corresponding author upon reasonable request.

## Abbreviations

AD: Alzheimer’s disease
AVLT: Auditory verbal learning test
BOLD: Blood-oxygenated level-dependent
CSF: Cerebrospinal fluid
DTI: Diffusion tensor imaging
FA: Fractional anisotropy
FC: Functional connectivity
fMRI: Functional magnetic resonance imaging
FU: Follow-up
FWHM: Full-width half-maximum
GLM: General Linear Model
ITT: Intention-to-treat
MCI: Mild cognitive impairment
MD: Mean diffusivity
NIBS: Non-invasive brain stimulation
PP: Per-protocol
ROI: Region of interest
SCD: Subjective cognitive decline
tDCS: Transcranial direct current stimulation
TE: Echo time
TR: Repetition time
WMT: Wiener Matrices test
