# Supplementary material for: Cognitive training and brain stimulation in patients with cognitive impairment: a randomized controlled trial
Source: Alzheimers Res Ther. 2024 Jan 11;16:6. doi: 10.1186/s13195-024-01381-3 (PMC10782634; doi:10.1186/s13195-024-01381-3)
Supplement: Supplementary file 1 — Additional file 1: Table S1. Baseline characteristics. Figure S1. Forest plot for performance outcomes (ITT sample). Intention-to-treat analyses of training and transfer effects at post and follow-ups. Abbreviations and units: Letter Updating # correct. Markov, % optimal actions. N-back, % correct and d-prime. WMT, % correct. VLMT (German version of the Auditory Verbal Learning Test, AVLT) # words recalled. Separate linear mixed model analyses were conducted for post-assessment and follow-up time points, for each task (i.e., 1/7-months FU values are derived from the same models as for the corresponding overall FU scores). In case of missing data, results are based on multiple imputation. FU, follow-up. WMT, Wiener Matrices Test. VLMT, verbal learning and memory test. For separate time points: N = 46 if not indicated otherwise. *n = 45. #n = 44. §n = 34. °n = 33. Table S2. Microstructural and volumetric analysis (MRI sample). Table S3. Self-reported incidence of adverse events (at least moderate symptoms). Table S4. Number of participants by group assignment and guess. [file 13195_2024_1381_MOESM1_ESM.docx]

**Supplementary Methods**

For the primary outcome (performance in the letter updating task at post-assessment), a linear mixed model (random intercept model) was conducted with the between-subject factor stimulation group (tDCS, sham) as covariate, including all time points, adjusted for baseline performance, age (stratification variables for randomization), and sex. Time-dependent trends were tested with a continuous time variable (centered) for training days and an additional quadratic time (centered) term to account for a curvilinear time trend. To model differences in changes over time between groups, we also included the interaction term group*time.

For the secondary outcome Markov decision task performance, the analysis was conducted in an identical manner.

To evaluate long term effects of outcomes, we used linear mixed models with outcome measures at post, 1 month and 7 months follow-up, adjusted for baseline of letter updating performance, baseline of the specific outcome, sex and age.

For the N-back task where we had two outcomes (two different levels of difficulty) for each participant for post intervention (1-back and 2-back), we used a generalized estimating equations model to achieve numeric stability of the model and to account for the repeated measures (factor load). We adjusted this model for age and baseline scores (measure for N-back was exponentiated by 4 to avoid skewness).

For evaluation of the treatment effect at post-assessment only (without measures at training days), linear models were computed adjusted for age, sex and baseline scores (for WMT and VLMT).

For ITT analysis, multiple imputation by chained equations was performed with 30 imputed datasets using predictive mean matching to estimate missing values. The following variables were included in the imputation model of training data: sex, age, stimulation condition, education, baseline letter updating performance, letter updating performance over all time points, baseline Markov task performance, Markov task performance over all time points.

**Supplementary Table 1.** Baseline characteristics.

|  | | | ITT | | | | PP | | | |
| --- | --- | --- | --- | --- | --- | --- | --- | --- | --- | --- |
|  | | | Total | Target group | Control group | p-value | Total | Target group | Control group | p-value |
| N (female) | | | 46 (18) | 21 (8) | 25 (10) |  | 39 (15) | 16 (6) | 23 (9) |  |
| N MCI/SCD | | | 39/7 | 20/1 | 19/6 |  | 32/7 | 15/1 | 17/6 |  |
| Age (years) | | | 70.0 (5.1) | 70.2 (5.6) | 69.7 (4.7) | 0.736 | 70.0 (5.2) | 70.2 (6.2) | 69.8 (4.6) | 0.836 |
| Education (years) | | | 15.3 (2.4) | 15.4 (2.6) | 15.2 (2.1) | 0.842 | 14.9 (2.3) | 14.6 (2.3) | 15.2 (2.2) | 0.412 |
| APOE e4 (N) | | | 19 | 8 | 11 | 0.586 | 17 | 10 | 7 | 0.509 |
| GDS score | | | 2.1 (1.0) | 2.3 (1.1) | 2.0 (0.8) | 0.201 | 2.2 (1.0) | 2.6 (1.1) | 2.0 (0.8) | 0.056 |
| CERAD scores, mean (SD) | | | | | | | | | | |
|  | Phonemic fluency (S words) | | 14.2 (4.0) | 14.6 (3.9) | 13.8 (4.2) | 0.543 | 13.8 (3.9) | 13.7 (3.9) | 13.8 (4.0) | 0.926 |
|  | BNT | | 14.2 (0.9) | 14.1 (0.8) | 14.2 (0.9) | 0.583 | 14.2 (0.9) | 14.2 (0.8) | 14.2 (1.0) | 0.917 |
|  | Figure copying retrieval | | 8.5 (2.3) | 8.4 (2.1) | 8.5 (2.6) | 0.991 | 8.4 (2.4) | 8.2 (2.1) | 8.5 (2.7) | 0.760 |
|  | Word list learning | | 17.7 (4.2) | 17.5 (4.2) | 17.9 (4.3) | 0.780 | 17.8 (4.1) | 17.2 (4.6) | 18.2 (3.8) | 0.478 |
|  | Word list retrieval | | 5.5 (2.1) | 5.4 (2.0) | 5.6 (2.2) | 0.784 | 5.6 (2.1) | 5.3 (2.2) | 5.7 (2.0) | 0.535 |
|  | Word list recognition | | 8.1 (4.4) | 8.4 (4.3) | 7.8 (4.6) | 0.617 | 7.9 (4.8) | 8.1 (4.9) | 7.8 (4.8) | 0.829 |
| Study-specific cognitive measures at baseline, mean (SD) | | | | | | | | | |  |
|  | LU | | 3.5 (2.8) | 3.0 (3.0) | 3.9 (2.7) | 0.275 | 3.5 (3.0) | 2.9 (3.3) | 4.0 (2.8) | 0.278 |
|  | Markov, % optimal actions | | 0.5 (0.1) | 0.5 (0.1) | 0.4 (0.1) | 0.363 | 0.4 (0.1) | 0.5 (0.1) | 0.4 (0.2) | 0.538 |
|  | N-Back | |  |  |  |  |  |  |  |  |
|  |  | % correct | 75.6 (17.9) | 73.1 (17.9) | 77.6 (18.1) | 0.398 | 76.0 (18.4) | 71.3 (19.1) | 79.3 (17.5) | 0.184 |
|  |  | d-prime | 1.9 (0.7) | 1.8 (0.5) | 1.9 (0.8) | 0.748 | 1.9 (0.7) | 1.8 (0.6) | 1.9 (0.8) | 0.474 |
|  | VLMT recall | | 9.1 (4.1) | 8.5 (3.4) | 9.6 (4.7) | 0.363 | 9.1 (4.1) | 8.0 (3.4) | 9.9 (4.4) | 0.160 |
|  | WMT, % correct | | 35.9 (18.6) | 33.6 (19.6) | 37.8 (17.9) | 0.454 | 6.4 (3.5) | 5.6 (3.8) | 7.0 (3.3) | 0.231 |

*Note.* ITT, Intention-to-treat. PP, per protocol. MCI, Mild cognitive impairment. SCD, Subjective cognitive decline. APOE, Apolipoprotein E. GDS, Geriatric depression scale (max. score: 15 points (cut off 6 points)). CERAD, Consortium to Establish a Registry for Alzheimer’s Disease. BNT, Boston Naming Test: maximal score 15. Figure copying retrieval: maximal score 11. Word list learning: maximal score 30. Word list retrieval: maximal score 10. Word list recognition: calculated by subtracting false positives from true positives, maximal score 10. LU, letter updating, maximal score 15. VLMT, verbal learning and memory test, maximal score 15. WMT, Wiener Matrices Test. p-values were derived from two-sided t-tests (from a χ^2^-test for APOE e4).

**
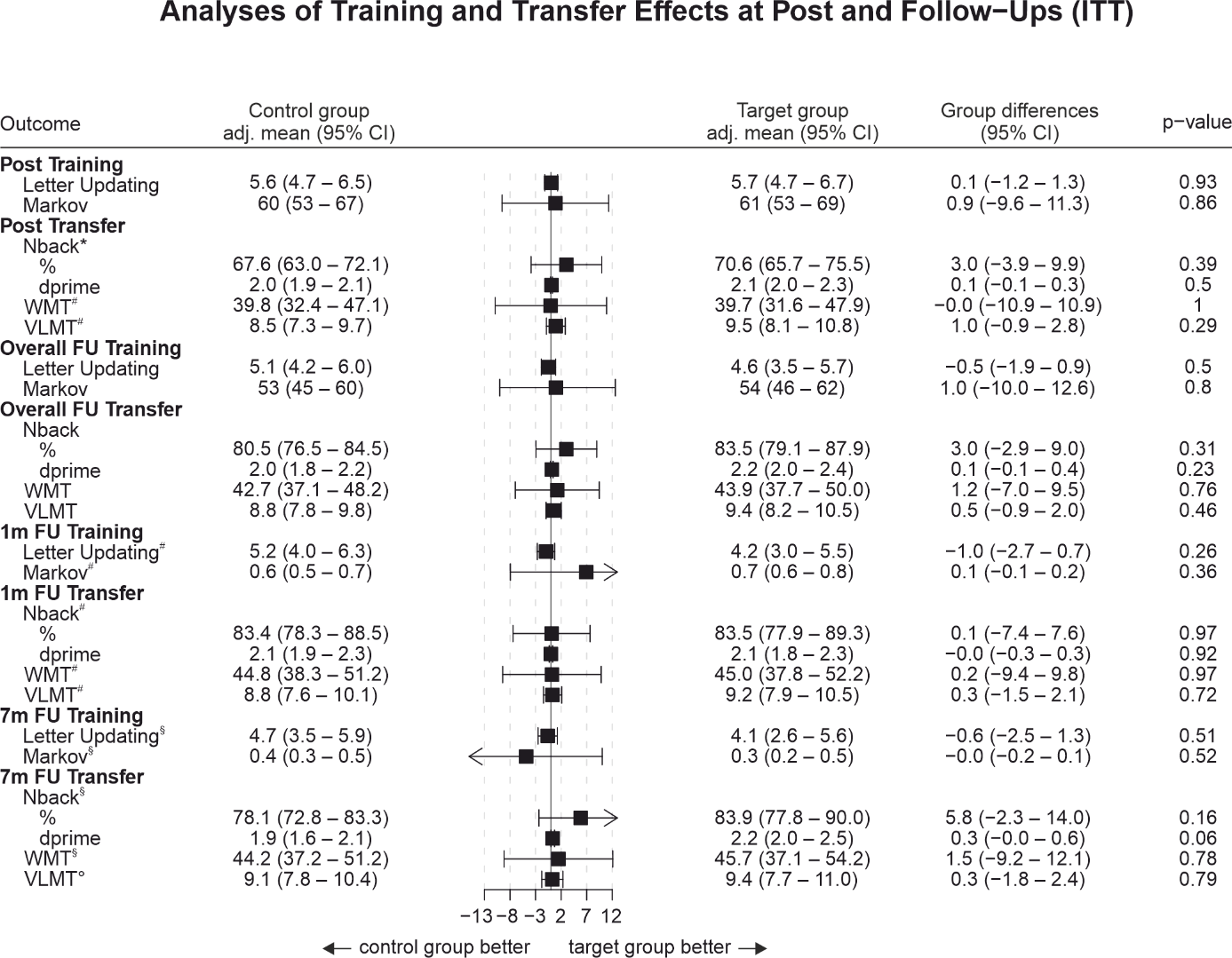
**

**Supplementary Figure 1. Forest plot for performance outcomes (ITT sample).** Intention-to-treat analyses of training and transfer effects at post and follow-ups. Abbreviations and units: Letter Updating # correct. Markov, % optimal actions. N-back, % correct and d-prime. WMT, % correct. VLMT (German version of the Auditory Verbal Learning Test, AVLT) # words recalled. Separate linear mixed model analyses were conducted for post assessment and follow-up time points, for each task (i.e., 1/7-months FU values are derived from the same models as for the corresponding overall FU scores). In case of missing data, results are based on multiple imputation. FU, follow-up. WMT, Wiener Matrices Test. VLMT, verbal learning and memory test. For separate time points: N = 46 if not indicated otherwise. *n = 45. ^#^n = 44. ^§^n = 34. °n = 33.

**Supplementary Table 2.** Microstructural and volumetric analysis (MRI sample).

|  | | Target group | | Control group | |
| --- | --- | --- | --- | --- | --- |
|  | | Pre | 7m FU | Pre | 7m FU |
| White matter in targeted pathways | | | | | |
|  | FA, mean (SD) | 0.31 (0.03) | 0.31 (0.03) | 0.31 (0.03) | 0.32 (0.03) |
|  | Volume, mean (SD) mm^3^ | 4127 (699) | 4018 (713) | 4099 (700) | 4011 (551) |
| Grey matter in the target region | | | | | |
|  | MD, mean (SD) x 10^3^ | 1.11 (0.07) | 1.09 (0.09) | 1.09 (0.10) | 1.13 (0.14) |
|  | Volume, mean (SD) mm^3^ x 10^2^ | 1.22 (1.19 x 10^3^) | 1.21 (9.16 x 10^4^) | 1.21 (6.90 x 10^4^) | 1.19 (7.43 x 10^4^) |

*Note.* FA. Fractional anisotropy. MD. Mean diffusivity.

**Supplementary Table 3**. Self-reported incidence of adverse events (at least moderate symptoms).

|  | | ITT | | | | PP | | | |
| --- | --- | --- | --- | --- | --- | --- | --- | --- | --- |
|  | | Total  N=46 | Target group  n = 21 | Control group  n = 25 | Incidence rate ratio for group differences  (95-% CI) | Total  N=39 | Target group  n = 16 | Control group  n = 23 | Incidence rate ratio for group differences  (95%-CI) |
| Observation time in days, mean (SD) | | 8.7 (0.8) | 8.7 (0.9) | 8.8 (0.7) |  | 9 (0) | 9 (0) | 9 (0) |  |
| Total number of adverse events | | 31/7.9  (5.4-11.0) | 15/8.2  (4.7-13.1) | 16/7.6  (4.5-12.0) | 1.1  (0.5-2.2) | 27/7.9  (5.3-11.3) | 15/9.8  (5.6-15.6) | 12/6.3  (3.4-10.6) | 1.5  (0.7-3.4) |
|  | Itching | 7/1.8  (0.8-3.5) | 4/2.2  (0.7-5.1) | 3/1.4  (0.4-3.7) | 1.5  (0.3-7.8) | 7/2  (0.9-4.0) | 4/2.6  (0.8-6.1) | 3/1.6  (0.4-4.1) | 1.6  (0.4-8.4) |
|  | Pain | 2/0.5  (0.1-1.6) | 2/1.1  (0.2-3.4) | 0 | .. | 2/0.5  (0.1-1.8) | 2/1.3  (0.2-4.0) | 0 | .. |
|  | Burning | 5/1.3  (0.5-2.7) | 4/2.2  (0.7-5.1) | 1/0.5  (0.0-2.1) | 4.6  (0.7-89.8) | 5/1.5  (0.5-3.1) | 4/2.6  (0.8-6.1) | 1/0.5  (0.0-2.3) | 4.9  (0.7-96.7) |
|  | Warmth/heat | 5/1.3  (0.5-2.7) | 2/1.1  (0.2-3.4) | 3/1.4  (0.4-3.7) | 0.8  (0.1-4.6) | 4/1.2  (0.4-2.7) | 2/1.3  (0.2-4.0) | 2/1.1  (0.2-3.3) | 1.2  (0.1-10.3) |
|  | Metallic/iron taste | 0 | 0 | 0 | .. | 0 | 0 | 0 | .. |
|  | Fatigue | 11/2.8  (1.5-4.8) | 2/1.1  (0.2-3.4) | 9/4.3  (2.1-7.7) | 0.3  (0.0-1.0) | 8/2.3  (1.1-4.4) | 2/1.3  (0.2-4.0) | 6/3.2  (1.3-6.4) | 0.4  (0.1-1.8) |
|  | Other | 1/0.3  (0.0-1.1) | 1/0.5  (0.0-2.4) | 0 | .. | 1/0.3  (0.0-1.3) | 1/0.7  (0.0-2.9) | 0 | .. |

*Note.* ITT, Intention-to-treat. PP, per protocol. Reported values are absolute frequency of the respective AEs / incidence rate per 100 patient days (95 % CI). For the ITT sample, 15 adverse events were self-reported by 4 participants in the target group and 16 adverse events were self-reported by 7 participants in the control intervention group. For the PP sample, 15 adverse events were self-reported by 4 participants in the target group and 12 adverse events were self-reported by 6 participants in the control intervention group.

**Supplementary Table 4.** Number of participants by group assignment and guess.

|  | | ITT | | | | PP | | | |
| --- | --- | --- | --- | --- | --- | --- | --- | --- | --- |
| Assignment |  | Response | | | | Response | | | |
|  |  | Target | Control | DK | Total | Target | Control | DK | Total |
|  | Target | 7 | 2 | 8 | 17 | 7 | 1 | 7 | 15 |
|  | Control | 6 | 4 | 13 | 23 | 6 | 3 | 12 | 21 |
|  | Total | 13 | 6 | 21 | 40 | 13 | 4 | 19 | 36 |

*Note.* ITT, Intention-to-treat. PP, per protocol. DK denotes “Don’t know”.
